# Social prescribing and students: A scoping review protocol

**DOI:** 10.1371/journal.pone.0289981

**Published:** 2023-08-17

**Authors:** Caitlin Muhl, Stephanie Wadge, Tarek Hussein

**Affiliations:** 1 Canadian Social Prescribing Student Collective, Toronto, Ontario, Canada; 2 School of Nursing, Faculty of Health Sciences, Queen’s University, Kingston, Ontario, Canada; 3 Faculty of Applied Health Sciences, Brock University, St. Catharines, Ontario, Canada; 4 Leslie Dan Faculty of Pharmacy, University of Toronto, Toronto, Ontario, Canada; Xiamen University - Malaysia Campus: Xiamen University - Malaysia, MALAYSIA

## Abstract

Across the globe, student champions are building the social prescribing student movement. Given the numerous linkages between social prescribing and students, there is a need to understand the extent and type of evidence on social prescribing and students. Doing so will address an important gap in the literature, as there are no evidence reviews on this topic. Thus, this scoping review aims to understand the extent and type of evidence on social prescribing and students. This review will be conducted in accordance with the JBI methodology for scoping reviews and will be reported in line with the Preferred Reporting Items for Systematic reviews and Meta-Analyses extension for Scoping Reviews (PRISMA-ScR). The search strategy will aim to locate both published and unpublished literature. No language or date restrictions will be placed on the search. The databases to be searched include MEDLINE (Ovid), CINAHL (EBSCO), Embase (Ovid), PsycINFO (Ovid), AMED (Ovid), ASSIA (ProQuest), Sociological Abstracts (ProQuest), Global Health (Ovid), Web of Science (Clarivate), Epistemonikos, JBI EBP Database (Ovid), and Cochrane Library. Sources of gray literature to be searched include Google, Google Scholar, Social Care Online (Social Care Institute for Excellence), SIREN Evidence and Resource Library (Social Interventions Research and Evaluation Network), and websites of social prescribing organizations and networks. Additionally, a request for evidence sources will be sent out to members of the Global Social Prescribing Student Council. Two independent reviewers will perform title and abstract screening, retrieval and assessment of full-text evidence sources, and data extraction. Data analysis will consist of basic descriptive analysis. Results will be presented in tabular and/or diagrammatic format alongside a narrative summary.

## Introduction

The social prescribing movement is creating a paradigm shift in our understanding of how to achieve health and wellbeing by shifting the conversation from ‘what is the matter *with* you’ to ‘what matters *to* you’ [1]. Social prescribing is “a means for trusted individuals in clinical and community settings to identify that a person has non-medical, health-related social needs and to subsequently connect them to non-clinical supports and services within the community by co-producing a social prescription–a non-medical prescription, to improve health and wellbeing and to strengthen community connections” [2 p. 9]. Altogether, there are over 20 countries involved in the social prescribing movement, and this number is rapidly increasing [1].

Across the globe, student champions are supporting this work by building the social prescribing student movement. There are student groups in Australia, Canada, Japan, Portugal, Singapore, the United Kingdom, and the United States [3], and this list continues to grow. Collectively, this global network of student champions has developed the Social Prescribing International Student Movement Framework [3] and established the Global Social Prescribing Student Council. In Canada, the Canadian Social Prescribing Student Collective was established in 2022 [4]. Our mission is to build the social prescribing student movement across Canada. As the Canadian Social Prescribing Student Collective Research Working Group, our aim is to build the evidence base around social prescribing. Given the focus of our group, we are particularly interested in the multiple intersections and reciprocal influence between social prescribing and students.

Students have a critical role to play in shaping the wider social prescribing movement [3]. Indeed, students are able and willing to meaningfully contribute to social prescribing research, policy, and practice, such as supporting efforts to generate robust evidence on social prescribing, assisting with policy development around social prescribing, or taking on the connector role in social prescribing programs–the sky is the limit when it comes to how students can engage.

Students also stand to benefit from social prescribing. Even before the COVID-19 pandemic, students were experiencing high rates of mental health challenges [5, 6]. As a result of the pandemic, students are dealing with increasing levels of housing and food insecurity, financial hardships, and lack of social connectedness and sense of belonging, which not only negatively impacts student health and wellbeing but also academic performance [6]. It is apparent that upstream approaches are needed to meet the needs of this population [7]. This suggests that social prescribing may have a key role to play in supporting student health and wellbeing.

It is also necessary to consider the importance of this topic in relation to the global healthcare crisis. As tomorrow’s leaders, students need to be equipped with the necessary tools to address this seemingly insurmountable challenge [3]. Social prescribing is one such tool that shows promise to transform health systems across the globe [1]. This points to the need to educate today’s learners on this tool to ensure that tomorrow’s leaders have it in their toolbox.

Given the numerous linkages between social prescribing and students, there is a need to understand the extent and type of evidence on social prescribing and students. A preliminary search of PROSPERO, MEDLINE (Ovid), JBI EBP Database (Ovid), Cochrane Library, Google, and Google Scholar was conducted on April 12, 2023. Only one underway evidence review on social prescribing and students was found [8], which is narrowly focused on enhancing student wellbeing through social prescribing. Notably, no evidence reviews with an aim to understand the extent and type of evidence on social prescribing and students were found. This calls for a review of the evidence on this topic.

There are several different types of evidence reviews [9]. Scoping reviews are conducted to examine the scope of a body of literature on a specific topic [9–12]. It is apparent that this is the most appropriate method to map the evidence on this topic since this type of review is useful for reviewing evidence in emerging fields or topics and for addressing broad review questions [10–12].

This scoping review aims to understand the extent and type of evidence on social prescribing and students. Knowledge gaps in the evidence base will be identified. The implications of the findings for the social prescribing student movement and the wider social prescribing movement will be discussed.

### Review questions

1. What is the extent and type of evidence on social prescribing and students?
2. What are the knowledge gaps in the evidence base around social prescribing and students?

### Eligibility criteria

#### Participants

This review will consider evidence sources with participants who are labelled as students. Evidence sources with participants who are not labelled as students will be excluded. Age will not be considered–eligibility will be determined solely based on the use of the term ‘student’.

#### Concept

This review will consider evidence sources that explore a concept that meets the following operational definition of social prescribing, even if it is not labelled as social prescribing:

Social prescribing is “a holistic, person-centred, and community-based approach to health and wellbeing that satisfies Condition 1 and either Condition 2 or Conditions 3 and 4:

- Condition 1: Identifier identifies that person has non-medical, health-related social needs (e.g., issues with housing, food, employment, income, social support)
- Condition 2: Identifier connects person to non-clinical supports and services within the community by co-producing a non-medical prescription
- Condition 3: Identifier refers person to connector
- Condition 4: Connector connects person to non-clinical supports and services within the community by co-producing a non-medical prescription” [2 p. 9].

Evidence sources that explore a concept that does not meet this definition will be excluded.

#### Context

This review will consider evidence sources from any context.

#### Types of evidence sources

This review will consider both published and unpublished literature. Evidence sources with quantitative, qualitative, and mixed methods study designs will be considered. In addition to primary research, this review will consider reviews and meta-analyses, theses and dissertations, and reports. Evidence sources without full text, text and opinion papers, and protocols will be excluded.

## Methods

This protocol has been registered on Open Science Framework (osf.io/m3ura) and published in this journal to promote transparency. This protocol was developed according to best practice guidance for scoping review protocols from the JBI Scoping Review Methodology Group [13]. This review will be conducted in accordance with the JBI methodology for scoping reviews [11] and will be reported in line with the Preferred Reporting Items for Systematic reviews and Meta-Analyses extension for Scoping Reviews (PRISMA-ScR) [14].

### Search strategy

The search strategy will aim to locate both published and unpublished literature. An initial limited search of MEDLINE (Ovid) and CINAHL (EBSCO) was undertaken to identify evidence sources on the topic. The text words contained in the titles and abstracts of relevant evidence sources, and the index terms used to describe the evidence sources, were used to develop a full search strategy for MEDLINE (Ovid) in Table 1. The search strategy was developed in consultation with an academic health sciences librarian. The research team will adapt the search strategy, including all identified keywords and index terms, for each included database and source of grey literature. This will be done in consultation with the academic health sciences librarian. The reference lists of all included evidence sources will be screened for additional evidence sources. No language or date restrictions will be placed on the search. In the event that translation becomes necessary, the review team will use DeepL Translator (DeepL SE, Cologne, Germany).

**Table 1:** Search strategy for MEDLINE (Ovid).

Number	Query	Results
1	((social or non-medical or non-clinical or community) adj (prescri* or referral*)).ti.	402
2	((social determinant* or social risk* or social need*) and (identif* or screen* or connect* or referral* or address* or navigat*)).ti.	755
3	1 or 2	1153
4	exp Students/	165205
5	student*.mp.	393326
6	4 or 5	393326
7	3 and 6	78

Search conducted on April 18, 2023

The databases to be searched include MEDLINE (Ovid), CINAHL (EBSCO), Embase (Ovid), PsycINFO (Ovid), AMED (Ovid), ASSIA (ProQuest), Sociological Abstracts (ProQuest), Global Health (Ovid), Web of Science (Clarivate), Epistemonikos, JBI EBP Database (Ovid), and Cochrane Library. Sources of gray literature to be searched include Google, Google Scholar, Social Care Online (Social Care Institute for Excellence), SIREN Evidence and Resource Library (Social Interventions Research and Evaluation Network), and websites of social prescribing organizations and networks, including the Social Prescribing Network, the Social Prescribing Youth Network, the Global Social Prescribing Alliance, the National Academy for Social Prescribing, and the Canadian Institute for Social Prescribing. Additionally, a request for evidence sources will be sent out to members of the Global Social Prescribing Student Council.

### Evidence source selection

Following the search, all identified evidence sources will be collated and imported into Covidence (Veritas Health Innovation, Melbourne, Australia) and duplicates removed. Following a pilot test, titles and abstracts will then be screened by two independent reviewers for assessment against the inclusion criteria for the review. Potentially relevant evidence sources will be retrieved in full, imported into Covidence, and assessed in detail against the inclusion criteria by two independent reviewers. Reasons for exclusion of evidence sources at full text that do not meet the inclusion criteria will be recorded and reported in the scoping review. Any disagreements that arise between the reviewers at each stage of the selection process will be resolved through discussion or with a third reviewer. The results of the search and the evidence source inclusion process will be reported in full in the final scoping review and presented in a PRISMA-ScR flow diagram [14].

### Data extraction

Data will be extracted from evidence sources included in the scoping review by two independent reviewers using a data extraction tool developed by the research team. The data extracted will include specific details about the objective, participants, concept, context, methods, and key findings relevant to the review questions. A draft data extraction tool is provided in Table 2. This tool will be modified and revised as necessary during the process of extracting data from each included evidence source. Modifications will be detailed in the scoping review. Any disagreements that arise between the reviewers will be resolved through discussion or with a third reviewer. Authors of evidence sources will be contacted to request missing or additional data, where required.

**Table 2:** Draft data extraction tool.

Citation Details	Objective	Participants	Concept	Context	Methods	Key Findings
						

### Data analysis and presentation

Data analysis will consist of basic descriptive analysis. Results will be presented in tabular and/or diagrammatic format in a manner that aligns with the review objective. A narrative summary will accompany the tabulated and/or charted results and will describe how the results relate to the review objective and questions.

## Supporting information

S1 Appendix Recommended items to address in a scoping review protocol*. (PDF)
